# COVID-19 Pandemic and Food Insecurity among Pregnant Women in an Important City of the Amazon Region: A Study of the Years 2021 and 2022

**DOI:** 10.3390/ijerph21060710

**Published:** 2024-05-30

**Authors:** Maria Tamires Lucas dos Santos, Kleynianne Medeiros de Mendonça Costa, Alanderson Alves Ramalho, João Rafael Valentim-Silva, Andreia Moreira de Andrade

**Affiliations:** 1 Graduate Program in Public Health, Multidisciplinary Center, Federal University of Acre, Cruzeiro do Sul 69980-000, AC, Brazil; 2 Multidisciplinary Center, Federal University of Acre, Cruzeiro do Sul 69980-000, AC, Brazil; kleynianne.costa@ufac.br; 3 Graduate Program in Public Health, Federal University of Acre, Rio Branco 69920-900, AC, Brazil; alanderson.ramalho@ufac.br (A.A.R.); andreia.andrade@ufac.br (A.M.d.A.); 4 Education and Technology College of Amazon, University of Vassouras, Saquarema 28990-720, RJ, Brazil; p.jrvalentim@gmail.com; 5 Laboratory of Biosciences of Human Motricity, Federal University of State of Rio de Janeiro, Rio de Janeiro 22290-240, RJ, Brazil; 6 Laboratory of Cineantropometry and Human Performance, Federal University of Santa Catarina, Florianópolis 88040-900, SC, Brazil

**Keywords:** food security, pregnancy, maternal nutrition, public health surveillance, cross-sectional studies

## Abstract

Introduction: Food insecurity (FI) experienced during pregnancy represents a relevant public health problem, as it negatively affects maternal and child health. Objective: To investigate the prevalence of FI among pregnant women during the COVID-19 pandemic and determine associated factors. Methods: A cross-sectional study was carried out in the period from 2021 to 2022, with a representative sample of 423 women resulting from a sample calculation based on the average (2912 births) that occurred in the years 2016 to 2020 in the only maternity hospital in the municipality. After analyzing the medical records, interviews were carried out with the postpartum women using a standardized questionnaire and the Brazilian Food Insecurity Scale. Poisson regression with robust variance was used to calculate prevalence ratios and 95% confidence intervals to measure associations. Results: FI was observed in 57.0% of cases and was associated with age under 20 years (PR = 1.52; 95% CI 1.29; 1.79), receipt of government assistance (PR = 1.31; 95% CI 1.10; 1.55), loss of family employment (PR = 1.40; 95% CI 1.20; 1.64), greater number of residents (PR = 1.17; 95% CI 1.00; 1.37), and prenatal care in a public institution (PR = 1.53; 95% CI 1.04; 2.26). Conclusion: There was a high prevalence of FI cases, associated with socioeconomic, demographic, and prenatal care characteristics during the COVID-19 pandemic.

## 1. Introduction

Food security (FS) ensures consistent access to nutritious food in adequate quantities without compromising other essential needs, while also respecting cultural differences and promoting sustainability across cultural, environmental, and economic domains. Conversely, the absence of this guarantee constitutes food insecurity (FI) [1]. This phenomenon is complex and multifactorial, intricately linked to precarious socioeconomic conditions. It poses a formidable global health challenge, detrimentally impacting nutrition and thereby compromising public health [2]. The experience of hunger epitomizes the most severe consequence of FI. Recent data suggest an alarming trend of hunger, with estimates indicating an escalation in malnutrition from 804 million in 2016 to 821 million people in 2017, translating to approximately one in every nine individuals globally [3].

Although hunger is intertwined with FI, the latter is more complex and comprehensive, as it is concerned not only with the quantity or availability of food, but also with the factors that interfere with the quality and access to food. Understanding FI also means understanding the various mechanisms and determinants that lead to both its extreme, which is hunger, as well as adverse conditions in social, environmental, cultural, economic, and health fields [4,5]. These are the reasons that motivated us to investigate FI, rather than just hunger, which is even more dramatic for the health of pregnant women and newborns.

With the onset of the COVID-19 pandemic in 2020, discourse and surveillance regarding hunger and FI intensified globally. “The State of Food Security and Nutrition in the World” (FSNW) report evaluated the pandemic’s repercussions, revealing that 828 million people grappled with hunger in 2021. Additionally, over 2.3 billion individuals experienced varying degrees of FI, accounting for nearly 30% of the world’s population. This dire scenario necessitates urgent attention, with warnings issued about the formidable challenges of rectifying it by 2030 [4]. In this context, the National Survey on FI in the Context of the COVID-19 Pandemic in Brazil (IVIGISAN) underscored that by the end of 2020, the nation had regressed to hunger levels reminiscent of those observed in 2004 [6].

The latest edition of the survey—II VIGISAN—unveiled a troubling escalation in hunger and FI among Brazilians, surging from 9% (19.1 million) in 2020 to just over 15% (33.1 million) in 2022, signifying an increase of approximately 14 million individuals. FI afflicted more than half of the country’s population (58.7%—125.2 million), with moderate and severe forms disproportionately affecting residents in the northern and northeastern regions. These regional disparities are rooted in historical socioeconomic factors, exacerbating vulnerabilities among marginalized communities [7].

The COVID-19 pandemic significantly impacted food security. The economic crisis triggered by the situation, along with isolation measures, travel restrictions, and border closures, resulted in disruptions in the supply chain [8,9]. Production, transportation, and distribution processes for food were compromised. Socioeconomic vulnerabilities were highlighted, and with the increase in inflation, families’ purchasing power diminished, leading to significant reductions in food consumption [9]. The economic crisis, combined with precarious living conditions and the strain on healthcare systems caused by the pandemic, had a major effect on ensuring the Human Right to Adequate Food (HRAF) [9,10].

The processes of food production and consumption within a society can profoundly affect environmental dynamics, the health–disease continuum, and social interactions. With the projected global population expected to increase by approximately 2 billion people over the next four decades, ensuring equitable food distribution and production poses a significant challenge [11,12].

During pregnancy, women’s vulnerability to FI is exacerbated by the physiological demands of the gestational process. With heightened nutritional requirements stemming from rapid cell growth and the development of new tissues and organs, ensuring adequate energy and nutrient intake among pregnant women is imperative. Failure to do so may precipitate a maternal–fetal conflict, compromising essential nutrients crucial for fetal development and maternal well-being [11]. Due to all the facts exposed here, FI experienced during pregnancy represents a public health problem that negatively affects maternal health, impacting mental health (with higher risks of stress, anxiety, and depression) [12,13,14,15]; nutritional health (increasing risks of anemia, as well as other complications such as obesity, diabetes, and hypertension) [16,17,18], which can present changes extending into the postnatal period [19]. Consequently, fetal and infant health may be affected, resulting in issues such as low birth weight, prematurity, congenital malformations, and developmental delays [12].

In the literature, the determinants of food insecurity are well-defined, with socioeconomic characteristics identified as primary drivers. However, scant attention is paid to vulnerable populations, such as pregnant women, particularly in the Amazon and northern regions of Brazil, underscoring the importance of this study. Monitoring food insecurity during pregnancy is pivotal for averting adverse outcomes and informing the formulation of targeted public health policies aimed at maternal and child welfare.

In the municipality of Cruzeiro do Sul, the second largest city in the state and the most important in the entire region, there have been no previous studies investigating food insecurity during pregnancy before the COVID-19 pandemic, which is dramatic. However, this adds novelty to our work. Nevertheless, a maternal–child cohort in Rio Branco, the largest city in the state, demonstrates a prevalence of food insecurity in pregnant women of 34.8% [20]. In our city, only one study investigated food insecurity during the pandemic period, but in a population of children under five years old, demonstrating a prevalence of 54% [21]. Globally, vulnerable groups such as women and pregnant women were impacted by the COVID-19 pandemic [7,22], which allows us to suggest that it may have exacerbated the situation for pregnant women in our region, giving significance to the present work.

Due to these facts, the hypothesis guiding our work is that the COVID-19 pandemic impacted the levels of food insecurity among pregnant women in our municipality. This is reinforced by studies showing that households headed by women are the most affected by food insecurity [7,23], demonstrating the need to investigate this issue with a focus on this group. Therefore, the objective of our study is to investigate the prevalence of food insecurity among pregnant women during the COVID-19 pandemic and to identify the associated factors.

## 2. Materials and Methods

### 2.1. Study Design

This study comprises an observational, analytical, and cross-sectional investigation conducted at the sole reference maternity hospital for the Juruá Valley region, situated in the municipality of Cruzeiro do Sul, Acre, in the Western Brazilian Amazon.

### 2.2. Characterization of the Collection Site

The state of Acre spans an area of 164,221.36 square kilometers and shares borders with Peru, Bolivia, and the Brazilian states of Amazonas and Rondônia [24]. Cruzeiro do Sul ranks as the second-largest municipality in Acre in terms of population, with 91,888 inhabitants, and is a part of the Juruá microregion, Tarauacá/Envira, alongside seven other municipalities. It boasts a population density of 10.46 inhabitants per square kilometer and a Human Development Index (HDI) of 0.664. The average monthly salary in the municipality stands at 1.8 times the minimum wage, with 44.2% of households earning monthly incomes of up to half a minimum wage [25].

Agriculture (corn, rice, banana), extractivism (açaí, buriti), fishing, and the production of cassava flour are the primary economic activities in the region. The region’s climate is characterized as hot and humid tropical, with an average annual temperature of 24 °C [26].

Primary healthcare is provided by 39 Family Health Teams, stationed across 25 Basic Health Units (BHUs), five annexes, and one Fluvial BHU. Additionally, the region is served by four multidisciplinary teams (e-Multi) and a Diagnostic Center, ensuring prenatal coverage of 99.4% and full family health team coverage. Specialized care is delivered through four institutions, including a General Hospital; an Emergency Care Unit (UPA), recently authorized to provide medium and high-complexity care; a Hospital for Sanitary Dermatology; and a maternity hospital (Hospital da Mulher e da Criança do Juruá), serving as a reference facility for the entire Juruá microregion, Tarauacá/Envira.

### 2.3. Population and Selection of the Census Group

The study population comprised puerperal women admitted to the Hospital da Mulher e da Criança Irmã Maria Inete Della Senta from 28 September 2021, to 1 January 2022. Over the period from 2016 to 2020, the institution recorded a total of 14,558 births per local occurrence, averaging 2912 births annually [27].

To determine the sample size, accounting for variations in the prevalence of FI across different regions of Brazil and the world, the scarcity of studies examining FI during pregnancy, particularly in the northern region, and the unique circumstances of the pandemic period, a sample of 408 puerperal women was selected. This calculation factored in an estimated prevalence of 50.0%, a margin of error of 5%, a confidence level of 95%, and a design effect of 1.0. Anticipating potential losses and refusals, the sample size was increased by 20.0%. The sample size calculation was conducted using the online version of OpenEpi version 3.01.

**Figure 1:**
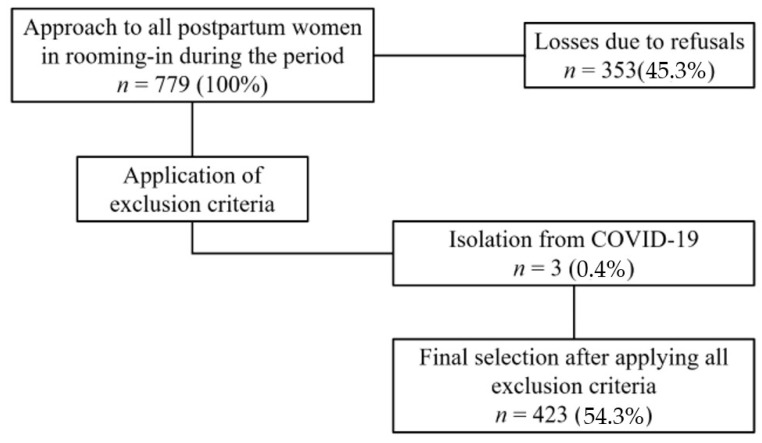
Census group selection.

All women admitted to the Joint Lodging of the Hospital da Mulher e da Criança Irmã Maria Inete Della Senta during the postpartum period within the study timeframe were initially considered for inclusion. However, women diagnosed with and isolated due to COVID-19, those with cognitive impairments, or individuals presenting clinical conditions precluding the administration of the research instrument until discharge, such as hemorrhages or eclampsia, were excluded. In this context, 779 postpartum women were initially approached, but 353 of these refused to participate in the study and 3 were excluded due to a diagnosis of COVID-19, resulting in a final sample of 423, according to the sample calculation. Figure 1 illustrates the flowchart depicting the selection process of the census group.

### 2.4. Data Collection Procedure

Initially, all researchers underwent training and standardization to ensure proficiency in completing the data collection form, processing identification, approaching participants, and administering the research instrument to the target audience. A pre-test involving 25 women was conducted to refine the research questionnaire, with none of these participants included in the final sample.

Women meeting the eligibility criteria received detailed explanations regarding the nature of the research, its objectives, methods, potential benefits, and risks involved. Volunteers provided their informed consent or assent forms if they were minors.

Subsequently, medical records, prenatal cards, and declarations of live births were consulted to extract clinical information, prenatal characteristics, birth details, obstetric complications, and newborn data. For this moment, a coded and standardized data collection script, printed on paper, was used. Subsequently, after a minimum recovery period of six hours postpartum for maternal well-being, interviews were conducted with the puerperal women using the face-to-face method, to validate sociodemographic information and administer the Brazilian Scale of FI (EBIA) to measure the outcome variable—FI. Data collection was conducted daily, during daylight hours, following a rotating schedule among researchers.

The EBIA comprises 14 structured binary (yes or no) questions aimed at identifying experiences of food insufficiency in the three months preceding its administration. Responses are scored (1 point for each affirmative response and 0 for negative responses) and subsequently summed and categorized based on the presence of minors under 18 years old in the household as follows: mild insecurity (1–5 points), moderate insecurity (6–10 points), and severe insecurity (11–14 points); and in the absence of minors under 18 years old as: mild insecurity (1–3 points), moderate insecurity (4–6 points), and severe insecurity (7–8 points) [28].

### 2.5. Exposure Variables

The exposure variables were categorized into three axes: sociodemographic characteristics, clinical and prenatal care characteristics, and childbirth and newborn characteristics, as outlined in references [29,30,31] and as displayed in Box 1.

Box 1 **Exposure variables according to their axes.**

Axis I—Sociodemographic Characteristics:

Age (≥20 and <20);

Education (>9 and ≤9);

Marital status (with or without partner);

Self-reported color (white; non-white);

Head of household (partner; woman; both; others);

Occupation (with or without remuneration);

Social class (Class A, B, and C (+3 salaries); Class D and E (up to 3 salaries));

Receipt of government assistance (no; yes);

Family job loss during the pandemic (no; yes);

Own home (no; yes);

Number of residents (≤4; >4);

Residential area (urban; rural)

Piped water (no; yes);

Electricity (no; yes);

Type of sewage in the residence (public sewage system; septic tank or rudimentary

none; open-air ditch; river or stream)

Axis II—Clinical and Prenatal and Birth Assistance Characteristics:

Type of prenatal care (private/mixed; or public)

Number of prenatal care (≥6; <6)

Childbirth method (cesarean section; vaginal)

Primiparity (no; yes);

Nutritional status in the last trimester of pregnancy (adequate; low weight; overweight and obesity)

Gestational diabetes (no; yes);

Increased blood pressure levels during pregnancy (no; yes);

Diagnosis of COVID-19 during pregnancy (no; yes);

Axis III—Newborn Characteristics:

Prematurity (no; yes);

Low birth weight (no; yes);

Macrosomia (no; yes);

Hospitalization in the Intensive Care Unit (ICU) (no; yes).

Social class definitions were based on economic criteria of minimum wage ranges, according to IBGE [31], divided into five categories: Class A (>15 salaries), Class B (5 to 13 salaries), Class C (3 to 5 salaries), Class D (1 to 3 salaries), and Class E (up to 1 salary). The reference for the minimum wage considered was the one in force for the year 2021 (BRL 1100.00). For maternal nutritional status classification, the Body Mass Index (BMI) was calculated from weight in kilograms divided by height in meters squared, verified at the end of gestation, and categorized according to gestational week; gestational diabetes was considered when fasting glucose was >92 mg/dl; blood pressure levels were considered increased during pregnancy when (systolic ≥ 140 mmHg and diastolic ≥ 90 mmHg) [29]; prematurity when birth occurred before 37 gestational weeks; low birth weight when the newborn’s weight was less than 2500 g; and macrosomia when weight was greater than 4000 g [30].

### 2.6. Data Analysis

Initially, reviews and consistency analyses were conducted on the database to make corrections where necessary. Missing values were treated as losses. Qualitative variables were described in the format of absolute and relative frequencies, and quantitative variables were expressed through measures of central tendency (mean, minimum, and maximum) and dispersion (standard deviation—SD). The associated factors were analyzed by Poisson regression with robust variance and adjusted for possible confounding factors. Eligible variables for entry into the adjusted model were those that in the crude analysis presented *p* < 0.20. The method of constructing the model used was the entry of variables, one by one, according to the increasing sequence of the *p*-value. Variables that in the adjusted model presented *p* < 0.05 were considered associated with the outcome. The adjustment of the final model was evaluated by the Omnibus test with a *p*-value less than 0.01 and by the Akaike Information Criterion (AIC).

The results were expressed in prevalence ratios (PRs), with their respective 95% confidence intervals (95% CIs). The analyses were performed using the Statistical Packages for the Social Sciences (SPSS) software package, version 26.0 from IBM.

## 3. Results

**Figure 2:**
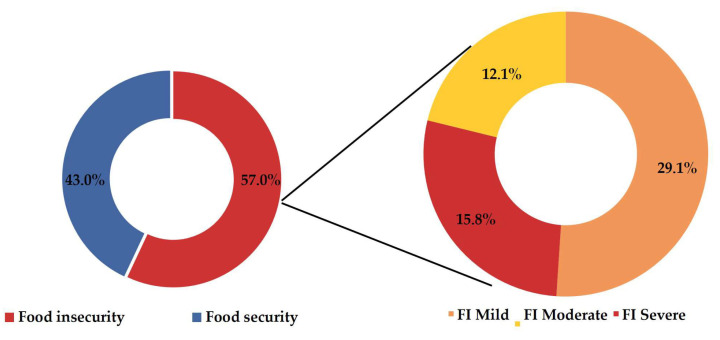
Prevalence of gestational FI (*n* = 423), during the COVID-19 pandemic, among women attended at a reference maternity hospital in Cruzeiro do Sul, Acre; Western Brazilian Amazon—2021.

A prevalence of gestational FI of 57.0% was observed, equating to 241 cases. Among these, 15.8% manifested the condition in its severe form, totaling 67 cases, as illustrated in Figure 2.

Participants had a mean age of 24.9 years (standard deviation = 6.7; minimum age of 13 and maximum of 44 years). A total of 16.8% (*n* = 71) were adolescents. In terms of education, the majority had completed high school (52.7%). Regarding marital status, 77.1% lived in a consensual union. In terms of ethnic-racial self-identification, the majority identified themselves as non-white (92.0%), with brown being the predominant color (77.1%). Approximately 48.7% had their partner as the head of the family, while 66.0% relied on an income of up to one minimum wage. Furthermore, 49.4% were beneficiaries of government aid, 61.9% resided in rural areas, and 72.6% were engaged in unpaid work. Regarding basic housing conditions, 48.0% had access to running water, and 88.4% (374) had access to electricity. It was observed that 35.0% of women did not have access to the sewage network, and the average number of residents in the household was 4.71 ± 1.96 (Table 1).

Regarding clinical and prenatal characteristics, it was observed that 87.2% of women received prenatal care at a public institution. The majority (58.6%) were multigravida, with an average gestational number of 2.51 (standard deviation = 2.09). Approximately 46.1% were overweight or obese at the end of pregnancy. Additionally, 9.0% were diagnosed with COVID-19, 15.4% were diagnosed with diabetes, and 16.8% had elevated blood pressure levels. The most prevalent delivery method was cesarean section, accounting for 67.4% of cases (Table 2).

Regarding newborns, the average weight was 3390 g (standard deviation = 0.509 g), with a minimum of 1070 g and a maximum of 4680 g. It was observed that 7.6% of babies were born prematurely, 6.1% had low birth weight, 6.6% had macrosomia, and 3.1% required admission to the Intensive Care Unit (ICU) (Table 2).

The analysis of factors associated with gestational food insecurity among women from a public maternity hospital in Cruzeiro do Sul, Acre, Western Amazon, Brazil, reveals significant socioeconomic and demographic influences (Table 3). Younger women, particularly those under 20, exhibited a higher prevalence of food insecurity compared to their older counterparts (*p* < 0.001). Educational attainment also played a crucial role, with women having nine years or less of education more likely to experience food insecurity (*p* = 0.001). Employment status was another critical factor, as women without paid employment faced higher food insecurity rates (*p* = 0.023). Social class disparities were evident, with women from lower social classes (D and E) being significantly more affected (*p* = 0.012). Government assistance recipients also showed higher food insecurity levels, underscoring the pandemic’s impact on economically vulnerable groups (*p* = 0.019, adjusted *p* = 0.002). Family job losses during the pandemic were strongly associated with increased food insecurity, further highlighting the economic strain on these households (*p* < 0.001). Larger households with more than four residents (*p* = 0.014, adjusted *p* = 0.047), rural residency (*p* = 0.023), and lack of electricity access (*p* = 0.003) were additional factors linked to higher food insecurity. Public prenatal care recipients (*p* = 0.001, adjusted *p* = 0.032) and those with fewer than six antenatal care visits (*p* = 0.009) were also more prone to food insecurity, indicating gaps in healthcare access and support. Finally, the method of childbirth influenced food insecurity prevalence, with vaginal deliveries associated with higher rates compared to cesarean sections (*p* = 0.007).

## 4. Discussion

The aim of this study was to investigate the prevalence of FI among pregnant women during the COVID-19 pandemic and determine associated factors. The main findings reveal that FI during pregnancy affected 57% of the participants, with 15.8% experiencing severe forms. Associated factors include being under 20 years of age, receiving government assistance, experiencing family job loss during the pandemic, living in households with more than four residents, and attending prenatal consultations in public health services. These results underscore the necessity for targeted interventions aimed at vulnerable groups, such as young pregnant women and families impacted by job loss and reliance on government assistance, to alleviate FI and promote maternal and fetal health during a pandemic.

Comparing our results with regional data, we observe that between 2017 and 2018, 36.7% of Brazilian households faced FI. In Acre, the prevalence was 58.5%, with 55.5% of cases classified as mild, 23.3% as moderate, and 21.2% as severe [28]. A study conducted in a mother–infant cohort in Rio Branco found a frequency of 34.8% of FI during pregnancy, with 24.6% mild, 4.8% moderate, and 5.4% severe [20]. Another study in São José dos Pinhais, Paraná, an important state in the south region of Brazil, identified a prevalence of 34.7% [32]. On the other hand, a study in Canada with pregnant women receiving prenatal care found a rate of 12.8% FI, while in Humadan, Iran, the prevalence was 67.0% [33]. The differing prevalences among pregnant women worldwide can be attributed to various measurement scales and the effects of sociodemographic, environmental, and dietary patterns [34].

Compared to pandemic period data, the prevalence in this study is lower than the frequency observed in the northern region (71.6%) and approaches the national average of Brazilian households (58.7%) [7]. Evaluating a cohort of 660 children in the Juruá Valley, FI was identified in 54% of households. Although this cohort consists of children under five years old, it is relevant to consider the possibility of many mothers skipping meals to feed their children, suggesting that if a child is food insecure, this may be the reality for all household residents [21].

This research also investigated factors associated with FI during pregnancy amid the COVID-19 pandemic. The results indicated that being under 20 years of age increased the risk of FI by over 50% compared to women aged 20 and older. This heightened risk may be linked to low education resulting from dropping out of school, difficulties in entering the job market, and financial dependence stemming from youth. All of these factors limit access to goods and services, contributing to high prevalence outcomes. It is noteworthy that young individuals are more likely to maintain inadequate eating habits, which, combined with the experience of FI, can elevate the risk of developing chronic diseases [35]. Ata from the II VIGISAN highlight that while food security is present in households comprised of adults, it is necessary to observe the family composition, as homes with economically dependent young individuals have a greater chance of experiencing FI. Furthermore, its presence in children and adolescents reveals negative effects on health and well-being conditions, which can compromise future physical and social potential [7].

Pregnant women who received government assistance and those whose family members lost their jobs during the pandemic had a higher prevalence of the outcome, which aligns with findings from other studies [20,36,37,38,39]. Programs such as family allowances and emergency aid, among others, appear to be insufficient to guarantee food security for families, especially in the context of a pandemic where purchasing power has been diminished due to inflation. While hunger tended to diminish when families had an income greater than the minimum wage per person, this was not guaranteed during the pandemic [7].

A greater number of residents in the household showed a positive association with FI, which can be partially explained by the need for a proportional increase in food to support the family [37,39]. The number of household residents has been identified in the literature as a factor associated with FI. The low level of access to food is more prevalent in families with three or more residents aged up to 18 years [7].

Providing prenatal care in the public network increased the prevalence of FI by more than 50%, in agreement with a study by [11] conducted in northeastern Brazil. The pursuit of prenatal care in public health services is predominantly undertaken by low-income women who face unmet basic needs. The risk of FI can be identified during the initial prenatal consultation, enabling pregnant women to receive nutritional support, enrollment in income transfer programs, and other strategies that ensure the right to adequate food [34].

Poor nutrition during pregnancy is an indicator that predisposes complications to maternal health, contributing to the development of depression, premature birth, increased risk of diabetes and hypertension, as well as adverse effects on childhood, such as an elevated risk of endocrine and cardiovascular diseases in infants, growth restriction, and low birth weight [40]. High-quality prenatal care combined with social protection strategies and food and nutrition policies are crucial for mitigating the detrimental effects of FI [38,41].

The high prevalence of severe FI identified in this study (15.8%) warrants attention, indicating a significant number of women experiencing hunger during pregnancy. This frequency represents approximately three times more than the national average of the population (4.6%) between 2017 and 2018 [28], and is also higher than the prevalence recorded in Rio Branco, Acre (5.4%) in 2015 [20]. However, it is lower than the rate observed in the northern region (25.7%) in a pre-pandemic period, and resembles estimates during the pandemic for Brazil (15.5%) [7], Latin America (14.2%), and the global population (11.7%) [41].

The escalation of FI and hunger has been a mounting concern since 2016, attributed to the weakening of public policies compounded by conflicts and climate change. This trajectory was accelerated with the emergence of the pandemic, which triggered significant social and economic upheavals. Social isolation negatively impacted economic activities, leading to a downturn in GDP, logistical challenges in food transportation, business closures, and a surge in unemployment, poverty, and hunger. With diminished purchasing power and heightened inflation, access to adequate food became even more constrained for the population. These findings underscore the pressing need for effective policies and interventions to combat FI and ensure the fundamental right to food for all individuals, particularly during crises like the COVID-19 pandemic [9,10,22,42].

The literature documents a wide variation in the prevalence of acute FI during pregnancy, ranging from 9.0% to 87.9%, with the highest rates reported in developing countries. In addition to the health consequences for pregnant women (anemia, diabetes, hypertension, obesity, postpartum depression, and suicide), gestational FI also impacts the newborn (congenital defects, low birth weight) [34,43], child development, and the long-term health of children [11,43,44].

Some measures already recognized in the literature can be strengthened to reduce the impacts of this nutritional problem on the health of pregnant women and their reproductive outcomes. Among good strategies are the early capture of these women, who can be screened during prenatal care; the promotion of education and nutritional counseling, which can influence pregnant women’s healthy food choices [34]; enrollment in income transfer programs, which increases the possibility of accessing more nutritious food [9]; strengthening iron and folate supplementation programs, which help prevent anemia [38]; strengthening local and family agriculture [45], with guarantees of production, transportation, and distribution, facilitating access to food, especially in geographically disadvantaged regions; as well as water access policies. The integration of care with interdisciplinary care increases the possibilities of meeting the needs of pregnant women. Finally, strengthening existing food and nutritional security policies in the face of constant dismantling [9,41] is also an important strategy for mitigating food insecurity during pregnancy.

Monitoring food insecurity is essential to highlight this nutritional health issue to society, the state, and to guide the governance of public policies for populations and territories with greater vulnerability, as is the case with Amazonian and northern Brazilian regions. Studies like this draw attention to the need for improvements in maternal and child healthcare policies and denounce structural issues of socioeconomic inequalities experienced by pregnant women, which hinder access to adequate nutrition and need to be further investigated. New studies need to be conducted. Longitudinal studies can be useful for analyzing and tracking the effects of food insecurity in post-pandemic times and for deepening understanding of its determinants during pregnancy. The impact of specific interventions can also be investigated to identify their effectiveness in the context of food insecurity in pregnant women.

Finally, certain limitations should be acknowledged. An important consideration is the cross-sectional design of the study, which prevents the establishment of causal relationships between the analyzed variables. It is crucial to emphasize that the primary objective of this study is to describe associations between factors and outcomes, rather than seeking cause-and-effect relationships. Additionally, there is the potential for information bias, particularly regarding participants’ perceptions of their own experiences as assessed by the EBIA scale. To mitigate these biases, researchers underwent thorough training, official sources of information were utilized whenever feasible, and the sample size was increased by 20% to compensate for any potential losses or refusals. It is essential to interpret the data cautiously, particularly given the atypical context of the pandemic, which exacerbated social and economic disparities, impacting access to food and potentially inflating the studied outcome. Lastly, the association of FI with socioeconomic, demographic, and prenatal care characteristics underscores the importance of assessing its prevalence in vulnerable populations such as pregnant women to inform public policies for maternal and child health.

## 5. Conclusions

Our results reveal an increase in food insecurity (FI) when compared to pre-pandemic data from regions within the same state and to pandemic data from the same municipality as this investigation, confirming our hypothesis that the pandemic has led to high levels of food insecurity in the region. The topic under study contributes to the field of public health, especially in the area of maternal and child health, as it portrays high rates of FI. Understanding the dynamics and magnitude of FI during pregnancy is crucial for healthcare professionals to recognize the need to screen these pregnant women early, identifying those with greater vulnerability and intervening so that they can have their right to adequate nutrition guaranteed. Assessing the risk of food insecurity should occur during prenatal care so that pregnant women receive nutritional support, are enrolled in income or food subsidy programs, and in other strategies that help mitigate this event during pregnancy to prevent adverse effects on the mother–child dyad.

Therefore, it is suggested that longitudinal studies can be useful to analyze and monitor the effects of food insecurity in post-pandemic times and to deepen understanding of its determinants during pregnancy. The impact of specific interventions can also be investigated to identify their effectiveness in the context of food insecurity in pregnant women.

## Figures and Tables

**Table 1:** Sociodemographic characteristics of women from a public maternity hospital in Cruzeiro do Sul, Acre—Western Amazon Brazil, 2021.

Variables/Categories		*n* = 423	% = 100
	Mean ± Standard Deviation	Min.	Max.
Age (Years)	24.87 ± 6.69	13	44
Number of residents	4.71 ± 1.96	2	14
Number of pregnancies	2.51 ± 2.09	1	19
Birth weight (Kg)	3.390 ± 0.509	1.070	4.680
		*n*	%
Education			
No education		7	1.7
Incomplete/Complete elementary education		124	29.3
Incomplete/Complete high school education		223	52.7
Higher education/Postgraduate degree		69	16.4
Marital status			
Single/Divorced/Separated		97	22.9
Married/Common-law marriage		326	77.1
Self-reported race			
White		34	8.0
Black		50	11.8
Brown/Mixed race		326	77.1
Yellow		6	1.4
Indigenous		7	1.7
Head of household			
Partner		206	48.7
Woman		61	14.4
Both (partner/woman)		75	17.7
Others		81	19.1
Occupation			
With remuneration		116	27.4
Without remuneration		307	72.6
Social Class ^a^			
Class A (+15 salaries)		4	0.9
Class B (5 to 15 salaries)		5	1.2
Class C (3 to 5 salaries)		24	5.7
Class D (1 to 3 salaries)		111	26.2
Class E (up to 1 salary)		279	66.0
Receipt of government assistance			
No		209	49.4
Yes		214	50.6
Housing Situation			
Own		335	79.2
Rented		35	8.3
Provided		53	12.5
Residential Zone			
Urban		161	38.1
Rural		262	61.9
Water Source			
Piped		203	48.0
Well		82	19.4
Cistern		95	22.4
Rivers or streams		43	10.2
Electricity			
No		49	11.6
Yes		374	88.4
Type of Sewage			
None		43	10.2
Public sewer system		79	18.7
Septic tank		144	34.0
Primitive septic tank		52	12.3
Open ditch/river or stream		105	24.8
Residents under 18 years old			
No		93	22.0
Yes		330	78.0

^a^ minimum wage in 2021 was BRL 1100.00.

**Table 2:** Clinical, prenatal, and newborn characteristics of women from a public maternity hospital in Cruzeiro do Sul, Acre, (*n* = 423)—Western Amazon, Brazil, 2021.

Variables/Categories	*n*	%
	423	100
Clinical and prenatal characteristics		
Type of prenatal care		
Mixed	37	8.7
Private	17	4.0
Public	369	87.2
Primiparity		
No	248	58.6
Yes	175	41.4
Childbirth method		
Cesarean section	285	67.4
Vaginal	138	32.6
Nutritional status in the last trimester of pregnancy (*n* = 421) ^a^		
Adequate	166	39.2
Low weight	60	14.2
Overweight and Obesity	195	46.1
Gestational diabetes		
No	287	67.8
Yes	71	16.8
Unknown	65	15.4
Increased blood pressure levels during pregnancy		
No	352	83.2
Yes	71	16.8
Diagnosis of COVID-19 during pregnancy		
No	385	91.0
Yes	38	9.0
Newborn characteristics		
Prematurity		
No	391	92.4
Yes	32	7.6
Low birth weight		
No	397	93.9
Yes	26	6.1
Macrosomia		
No	395	93.4
Yes	28	6.6
Hospitalization in the ICU ^b^		
No	410	96.9
Yes	13	3.1

^a^ Losses due to missing information; ^b^ ICU = Intensive Care Unit.

**Table 3:** Factors associated with gestational FI in women from a public maternity hospital in Cruzeiro do Sul, Acre, (*n* = 423) Western Amazon, Brazil, 2021.

Variables/Categories	Food Insecurity (*n* = 423)				Crude Analysis			Adjusted Analysis		
	No *n* (%)		Yes *n* (%)							
	182 (43)		241 (57)		PR ^a^	CI 95% ^b^	*p*-Value ^c^	PR ^a^	CI 95% ^b^	*p*-Value ^c^
Age							<0.001			<0.001
≥20	145	50.5	142	49.5	1.00	-		1.00	-	
<20	37	27.2	99	72.8	1.47	1.37–1.72		1.52	1.29–1.79	
Years of Education							0.001			
>9 years	140	47.9	152	52.1	1.00	-				
≤9 years	42	32.1	89	67.9	1.30	1.11–1.53				
Marital Status							0.160			
With partner	36	37.1	61	62.9	1.00					
Without partner	146	44.8	180	55.2	0.88	0.73–1.05				
Self-reported Race							0.895			
White	15	44.1	19	55.9	1.00	-				
Non-white	167	42.9	222	57.1	1.02	0.75–1.39				
Occupation							0.023			
With remuneration	61	52.6	55	47.4	1.00					
Without remuneration	121	39.4	186	60.6	1.28	1.03–1.58				
Social Class ^d^							0.012			
Class A, B, and C (more than 3 salaries)	23	69.7	10	30.3	1.00					
Class D and E (up to 3 salaries)	159	40.8	231	59.2	1.96	1.16– 3.30				
Receipt of government assistance							0.019			0.002
No	102	48.8	107	51.2	1.00	-		1.00	-	
Yes	80	37.4	134	62.6	1.22	1.03–1.45		1.31	1.10–1.55	
Family job loss during pandemic							<0.001			<0.001
No	159	47.7	174	52.3	1.00			1.00	-	
Yes	23	25.6	67	74.4	1.42	1.22–1.67		1.40	1.20–1.64	
Own domicile							0.040			
No	30	34.1	58	65.9	1.00	-				
Yes	152	45.4	183	54.6	0.83	0.69–0.99				
Number of residents							0.014			0.047
≤4 residents	117	48.1	125	51.9	1.00			1.00	-	
>4 residents	66	36.3	116	63.7	1.23	1.04–1.45		1.17	1.00–1.37	
Residential Zone							0.023			
Urban	81	50.3	80	49.7	1.00	-				
Rural	101	38.5	161	61.5	1.24	1.03–1.48				
Piped Water							0.473			
No	91	41.4	129	58.6	1.00	-				
Yes	91	44.8	112	55.2	0.94	0.80–1.11				
Electricity							0.003			
No	13	26.5	36	73.5	1.00	-				
Yes	169	45.2	205	54.8	0.75	0.62–0.90				
Type of sewage in the residence							0.007			0.064
Public sewerage system; Septic tank or rudimentary	131	47.6	143	52.4	1.00	-		1.00	-	
None; Open-air ditch; River or stream	51	34.5	97	65.5	1.25	1.06–1.47		1.16	0.99–1.36	
Type of prenatal care							0.001			0.032
Private or mixed	37	68.5	17	31.5	1.00	-		1.00	-	
Public	145	39.3	224	60.7	1.93	1.29–2.88		1.53	1.04–2.26	
Number of ANC ^e^							0.009			
≥6 ANC	152	46.1	178	53.9	1.00	-				
<6 ANC	30	32.3	63	67.7	1.26	1.06–1.49				
Childbirth method							0.007			
Cesarean section	135	47.4	150	52.6	1.00	-				
Vaginal	47	34.1	91	65.9	1.25	1.06–1.47				
Prematurity of the newborn							0.168			
No	164	41.9	227	58.1	1.00	-				
Yes	18	56.3	14	43.8	0.75	0.50–1.13				
Low birth weight							0.528			
No	171	42.6	230	57.4	1.00	-				
Yes	11	50.0	11	50.0	0.87	0.57–1.34				

^a^ PR = Prevalence ratio; ^b^ 95% CI = 95% confidence interval; ^c^ Wald teste for heterogeneity; ^d^ minimum wage in 2021 (BLR 1100.00); ^e^ ANC = antenatal care.

## Data Availability

Data available upon request—The data supporting the conclusions of this study are available upon reasonable request made to the authors. The data set is not publicly available as it details information that compromises the privacy of the research participants.
